# Reproducible proteomics sample preparation for single FFPE tissue slices using acid-labile surfactant and direct trypsinization

**DOI:** 10.1186/s12014-018-9188-y

**Published:** 2018-03-06

**Authors:** Melanie Christine Föll, Matthias Fahrner, Victor Oginga Oria, Markus Kühs, Martin Lothar Biniossek, Martin Werner, Peter Bronsert, Oliver Schilling

**Affiliations:** 1 Institute of Molecular Medicine and Cell Research, Faculty of Medicine, University of Freiburg, Stefan Meier Strasse 17, 79104 Freiburg, Germany; 2 Faculty of Biology, Albert-Ludwigs-University Freiburg, Freiburg, Germany; 3 Spemann Graduate School of Biology and Medicine (SGBM), Albert-Ludwigs-University Freiburg, Freiburg, Germany; 4 Institute for Surgical Pathology, Medical Center – University of Freiburg, Freiburg, Germany; 5 Comprehensive Cancer Center Freiburg, Medical Center – University of Freiburg, Freiburg, Germany; 6 Faculty of Medicine, University of Freiburg, Freiburg, Germany; 7 German Cancer Consortium (DKTK) and German Cancer Research Center (DKFZ), Heidelberg, Germany; 8 BIOSS Centre for Biological Signaling Studies, University of Freiburg, 79104 Freiburg, Germany

**Keywords:** Mass spectrometry, Proteomics, Label-free quantitation, FFPE, Archival tissue, FASP, RapiGest, Biomarker discovery, Stained tissue

## Abstract

**Background:**

Proteomic analyses of clinical specimens often rely on human tissues preserved through formalin-fixation and paraffin embedding (FFPE). Minimal sample consumption is the key to preserve the integrity of pathological archives but also to deal with minimal invasive core biopsies. This has been achieved by using the acid-labile surfactant RapiGest in combination with a direct trypsinization (DTR) strategy. A critical comparison of the DTR protocol with the most commonly used filter aided sample preparation (FASP) protocol is lacking. Furthermore, it is unknown how common histological stainings influence the outcome of the DTR protocol.

**Methods:**

Four single consecutive murine kidney tissue specimens were prepared with the DTR approach or with the FASP protocol using both 10 and 30 k filter devices and analyzed by label-free, quantitative liquid chromatography–tandem mass spectrometry (LC–MS/MS). We compared the different protocols in terms of proteome coverage, relative label-free quantitation, missed cleavages, physicochemical properties and gene ontology term annotations of the proteins. Additionally, we probed compatibility of the DTR protocol for the analysis of common used histological stainings, namely hematoxylin & eosin (H&E), hematoxylin and hemalaun. These were proteomically compared to an unstained control by analyzing four human tonsil FFPE tissue specimens per condition.

**Results:**

On average, the DTR protocol identified 1841 ± 22 proteins in a single, non-fractionated LC–MS/MS analysis, whereas these numbers were 1857 ± 120 and 1970 ± 28 proteins for the FASP 10 and 30 k protocol. The DTR protocol showed 15% more missed cleavages, which did not adversely affect quantitation and intersample comparability. Hematoxylin or hemalaun staining did not adversely impact the performance of the DTR protocol. A minor perturbation was observed for H&E staining, decreasing overall protein identification by 13%.

**Conclusions:**

In essence, the DTR protocol can keep up with the FASP protocol in terms of qualitative and quantitative reproducibility and performed almost as well in terms of proteome coverage and missed cleavages. We highlight the suitability of the DTR protocol as a viable and straightforward alternative to the FASP protocol for proteomics-based clinical research.

**Electronic supplementary material:**

The online version of this article (10.1186/s12014-018-9188-y) contains supplementary material, which is available to authorized users.

## Background

Human tissue specimens represent the most valuable material for translational clinical research e.g. for biomarker discovery and validation as well as for studying molecular disease pathways [1, 2]. For more than a century, pathologists have been preserving tissue specimens by formalin-fixation and paraffin-embedding (FFPE), a process which crosslinks biomolecules and dehydrates the specimens such as to prevent enzymatic degradation [3, 4]. Therefore, FFPE tissues can be long-term stored at room temperature without quality reduction, what has led to vast FFPE tissue archives in clinics that are often accompanied by clinical data like survival time and therapy response [5].

Several protocols successfully used FFPE tissues for bottom-up mass spectrometry based shot-gun proteomics [6–14]. This represents a significant leap forward in clinical proteomics. For a long time the highly crosslinked FFPE tissue specimens were considered to not be amenable to proteomic studies using liquid chromatography–tandem mass spectrometry (LC–MS/MS). However, Shi et al. [15] established heat induced antigen retrieval (HIAR) to remove formalin-induced crosslinks from proteins for immunohistochemical analyses. Since then, FFPE tissues are the standard material for immunohistological based clinicopathological diagnosis and also gained importance for biomarker studies [15]. HIAR enabled mass-spectrometry based proteomics studies of FFPE tissues with similar protein extraction efficiencies and numbers of identified peptides as observed for the first time by Hood et al. [13]. Many protocols for LC–MS/MS based studies of FFPE tissue specimens, use sodium dodecyl sulfate (SDS) in the extraction buffer. Being a strong detergent SDS is useful to solubilize and extract proteins from FFPE tissues, but it suppresses tryptic digestion and is incompatible with LC–MS/MS. Researchers have used diverse workflows to remove SDS. The most common is the filter aided sample preparation (FASP), in which SDS is successively exchanged with high molar urea in a centrifugal filter unit [16]. All SDS based protocols have in common that protein loss is inevitable due to the SDS cleanup step. This is typically offset by increasing the amount of input material.

There are several proteomic studies of FFPE samples which replace SDS extraction buffers with buffers that are compatible with subsequent trypsin digestion and LC–MS/MS analysis [5, 10, 11, 14, 17–21]. These protocols are called “direct trypsinization protocols”, because they proceed directly from HIAR and protein extraction to tryptic digestion, preventing sample loss due to SDS removal. The extraction buffers most routinely used for direct trypsinization protocols are 100 mM ammonium bicarbonate and 20% acetonitrile, the Liquid Tissue^®^ kit buffer or buffers with surfactants, such as RapiGest, which are compatible with trypsin digestion and mass-spectrometry measurement [10, 11, 13, 14, 20, 22]. RapiGest is an anionic, acid labile surfactant, which improves protein extraction and solubilization, but at the same time does not hinder tryptic digestion when used in a concentration of 0.1% [23]. Contrary, RapiGest was originally developed to enhance enzymatic digestion of proteins due to unfolding and solubilization hydrophobic proteins [23]. In addition to being helpful for protein extraction and tryptic digestion, RapiGest is easily degraded under acidic conditions yielding breakdown products that do not interfere with reversed phase chromatography. Accordingly, RapiGest is easily removed prior to LC–MS/MS analysis [23].

Two recent studies used a direct trypsinization approach with RapiGest (DTR) on small laser micro-dissected FFPE tissues [20, 21]. Azimi et al. used hematoxylin & eosin (H&E) stained FFPE cutaneous squamous cell carcinoma tissue to concisely collect tumor cells in the skin by laser microdissection. The obtained tissue was prepared using DTR and analyzed by LC–MS/MS [21]. Longuespee et al. applied a RapiGest buffer for the preparation of less than 3000 breast cancer cells obtained by laser microdissection from unstained FFPE tissue. An impressive number of 1400 proteins were identified and quantified by LC–MS/MS [20]. In order to perform accurate laser microdissection and for an exact differentiation between histological areas of interest (e.g. tumourus vs. non-tumourus regions) and non-relevant areas, tissue staining before laser microdissection represents an optimized solution.

The increasing prevalence of DTR-based proteomics of FFPE specimens motivated us to compare the DTR method (using a 0.1% RapiGest containing buffer) with the FASP approach for its performance and reproducibility. Furthermore, we investigated the compatibility of the standard histological tissue stainings H&E, hematoxylin and hemalaun on the DTR protocol, that we consider as a useful tool for studying small FFPE tissues in clinical proteomic research.

## Methods

### FFPE mouse kidney samples

Maintenance of animal strains and work performed in this study was carried out in accordance with institutional guidelines and the German law for animal protection (Tierschutzgesetz) as published on May 18th 2006 with last amendment on July 28th 2014. Ethics approval registration number is G14/18 RP regional council Freiburg. Kidneys of a male, 6-months old C57 black 6 mouse lacking cathepsin L (Ctsl^−/−^) were removed immediately after sacrificing it. Formalin fixation and paraffin embedding were performed as described previously [24]. 10 µm thick sections were sliced from one kidney FFPE tissue block using a microtome and mounted onto glass slides. Deparaffinization was performed by immersing the glass slides with the tissues three times 5 min into xylol. For rehydration the tissue was incubated for two times 5 min in 99% ethanol, following 20 s in 99, 96, 70 and 50% ethanol. The tissues were stored in distilled water until further proceeding. Twelve single kidney tissue slices with a tissue area of around 60 mm^2^, were transferred into separate reaction tubes and used as replicates for each of the three protocols (DTR, FASP 10 k, FASP 30 k).

### FFPE human tonsil samples

Patient consent was obtained from each patient before inclusion into this study. Tonsils were treated with formalin directly after surgical removal and embedded in paraffin as described previously [25]. 5 µm thick sections from two FFPE tonsil tissue blocks were sliced with a microtome and mounted onto glass slides. Deparaffinization and rehydration was performed according to a standard protocol [25]. For each tonsil, adjacent tissue slices were stained with hematoxylin, H&E, hemalaun or left unstained as control. For the hematoxylin staining the tissues were incubated for 4 min in hematoxylin solution modified acc. to Gill III and afterwards shortly washed with acetic acid in aqueous solution and furthermore, rinsed with water for about 5 min. The same procedure was performed for the H&E staining but afterwards the tissue was incubated in eosin for 1 min and rinsed again with water. Hemalaun staining was performed by immersing the tissue in Mayer’s acid Hemalaun solution for 1 min and rinse it with water for about 5 min.

The tissues were stored in distilled water until further proceeding. The 16 single deparaffinized and dehydrated tissue slices were transferred into separate reaction tubes with a scalpel. The tissue area for tonsil 1 was 118 mm^2^, for tonsil 2 93 mm^2^.

### Direct tissue trypsinization using a RapiGest containing buffer (DTR)

**Fig. 1:**
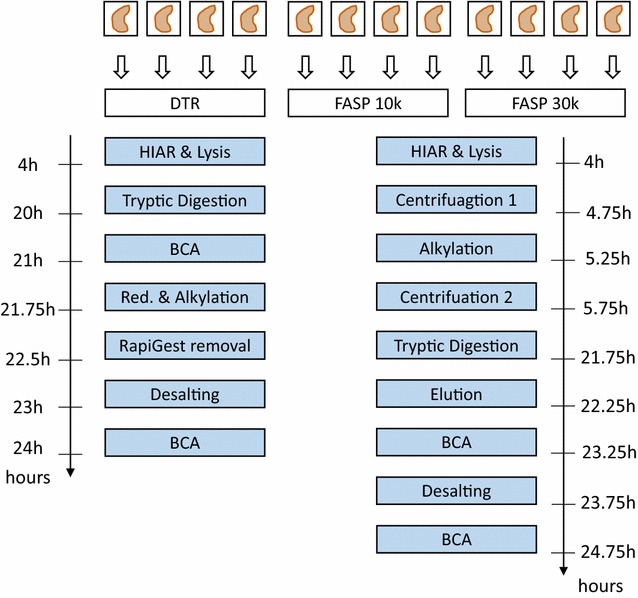
Schematic Workflow for the DTR versus FASP comparison. Four single, deparaffinized murine kidney FFPE tissues were separately prepared with each of the three sample preparation protocol. For the DTR protocol, a buffer containing 0.1% Rapigest in 0.1 M HEPES pH 8 and 1 mM DTT was used for heat-induced antigen retrieval (HIAR) and lysis of the tissue. As RapiGest is compatible with tryptic digestion, direct trypsinization is the key feature of the DTR protocol. RapiGest is later removed by acidifying the sample. Protein concentration is estimated to not overload the C18 stage tips during desalting step and later to inject the same amounts of peptides into the mass spectrometer. The FASP protocol makes use of a buffer containing 4% SDS, 0.1 M HEPES pH 7.5 and 0.05 M DTT. Before digestion the SDS is removed using centrifugal filter units with nominal molecular weight cut offs of either 10,000 or 30,000 Da. After digestion, the peptides are eluted from the filter units and desalted before mass-spectrometry analysis

The sample preparation steps for the DTR protocol are shown in Fig. 1. 100 µl (200 µl for the tonsil samples) of an aqueous buffer containing 0.1% RapiGest SF (Waters, Milford, MA, USA), 0.1 M HEPES pH 8 (AppliChem, Darmstadt, Germany) and 1 mM dithiothreitol (DTT) (AppliChem, Darmstadt, Germany) were added to each reaction tube, containing a single tissue slice. The buffered samples were incubated in a thermo shaker (TS1 ThermoShaker, Biometra, Göttingen, Germany) at 95 °C and 750 rpm for 4 h to perform heat induced antigen retrieval and protein extraction. The tonsil tissue was further homogenized in a biorupter (Diagenode) for 10 cycles (5 s on, 10 s off). The pH of each sample was checked and if necessary adjusted to pH 7–8. Sequencing grade trypsin (Worthington, Lakewood, NJ, USA) was added in a ratio of at least 2 µg per mm^3^ tissue. For trypsinization, the sample was incubated at 37 °C over night. In order to remove potential cell debris the samples were centrifuged at 19,000*g* for 15 min. Additional centrifugation was performed when cell debris were still present in the supernatant. The supernatant was transferred into a new reaction tube. If required, 2 × 5 µl were used to estimate the peptide concentration in the sample with the Pierce BCA protein assay kit (Thermo Scientific, Rockford, USA). The cysteine residues of the peptides in the supernatant were then reduced and alkylated by incubation in 10 mM DTT for 15 min at 37 °C, followed by 30 mM iodacetamide (Sigma-Aldrich, St. Louis, USA) for 15 min at 37 °C and again 10 mM DTT for 15 min at 37 °C. For RapiGest removal the samples were brought to a final concentration of 3 M guanidinium chloride (AppliChem, Darmstadt, Germany), acidified (pH < 3) with hydrochloric acid (Merck, Darmstadt, Germany) and incubated for at least 30 min at 37 °C. Any precipitate was removed by centrifugation at 19,000*g* for 10 min. 15 µg of peptides per sample were desalted using self-packed C18 STAGE tips (Empore, St. Paul, MN, USA) [26]. BCA assay was performed and 3 µg peptides per sample were vacuum dried in a centrifugal vacuum concentrator (Eppendorf, Hamburg, Germany) and stored at − 80 °C until measured by LC–MS/MS.

### Filter aided sample preparation (FASP)

Sample preparation was performed according to previously published protocols with minor adaptations [16, 27]. The workflow is depicted in Fig. 1. 10 and 30 k filter units were used for two independent FASP approaches called FASP 10 k and FASP 30 k, respectively. Heat induced antigen retrieval and protein extraction were achieved by boiling the kidney tissues in 100 µl of an aqueous buffer containing 4% sodium dodecyl sulfate (SDS) (Serva Electrophoresis GmbH, Heidelberg), 0.1 M Tris–HCl pH 7.6 and 0.1 M DTT for 4 h at 95 °C and 750 rpm. In order to remove SDS, the lysate was added to ultrafiltration devices (Microcon Ultracel YM-10 and YM-30 filtration devices, Merck Millipore, Darmstadt, Germany) which were pre-filled with 100 µl of freshly prepared 8 M urea buffer.

The SDS removal was performed as described in Nature protocols [16]. Trypsin digestion was performed by adding 40 µl ammonium bicarbonate buffer containing 2 µg sequencing grade trypsin (Worthington, Lakewood, NJ, USA). After digestion the peptides were eluted twice, using 40 µl ammonium bicarbonate by centrifugation at 14,000*g* for 15 min. The peptide concentration was estimated by using the Pierce BCA protein assay kit in triplicates. 12 µg of peptides were acidified with trifluoroacetic acid and desalted using self-packed C18 STAGE tips (Empore, St. Paul, MN, USA) [26]. Eluted peptide amounts were measured by BCA assay and 3 µg were vacuum dried in a centrifugal vacuum concentrator (Eppendorf, Hamburg, Germany) and stored at − 80 °C until measured by LC–MS/MS.

### LC–MS/MS analysis

Vacuum dried samples were dissolved in 40 µl aqueous buffer containing 2% acetonitrile and 0.3% acetic acid, sonicated for 5 min and transferred to the measurement tubes. 5 µl of each sample were analyzed by an Orbitrap Q-Exactive plus (Thermo Scientific) mass spectrometer coupled to an Easy nanoLC 1000 (Thermo Scientific) with a flow rate of 300 nl/min. Buffer A contained 0.3% (v/v) acetic acid and buffer B 0.3% (v/v) acetic acid in 80% acetonitrile. They were applied with an increasing gradient of acetonitrile over time (0–60% (v/v) acetonitrile in 90 min) in order to separate the peptides on the analytical column [Acclaim PepMap column (Thermo Scientific)], 2 µm particle size, 100 Å pore size, length 150 mm, inner diameter 50 µm). The MS was operated in data dependent mode and each MS scan was followed by a maximum of ten MS/MS scans. The mass range from 300 to 2000 Dalton was analyzed.

### Data analysis

MaxQuant (V1.5.2.8) software was used for data analysis [28]. Peptide identification was performed with the Andromeda search engine using human or mouse proteome databases containing reviewed Uniprot sequences without isoforms downloaded from Uniprot on 18th May 2015 (mouse, 16,711 entries) and 15th April 2016 (human, 20,193 entries). Decoys for the database search were generated with the revert function. The precursor mass tolerance for the initial search was 20 ppm and for the main search 4.5 ppm whereas the fragment mass tolerance was 20 ppm. Tryptic cleavage specificity with two missed cleavages was applied, minimal peptide length was set to seven amino acids and I = L was enabled. Carbamidomethyl at cysteines was the only fixed modification. The false discovery rate (FDR) for peptides and proteins was set to 0.01. Label-free quantitation (LFQ) on at least one peptide per protein was performed using the MaxLFQ algorithm and the re-quantify function [29]. The MaxQuant output was further processed in R (V3.3.1) with RStudio as an integrated development environment. Reverse and potential contaminant entries were removed. LFQ intensities were log2 transformed for plotting intensity distribution and calculating the Pearson correlation coefficient. Functional classification of the identified proteins was performed with PANTHER (V11.1) [30, 31]. The open, web-based platform galaxy [32, 33] was used to calculate the identified proteins’ molecular weight, isoelectric point and gravy score with biopython [34] as well as potential transmembrane domains with a hidden markov model [35, 36]. In case of peptides containing selenocysteine the single amino acid “U” was deleted in order to obtain a gravy score. In three replicates, there was one peptide containing three “B” and two “Z” amino acids, in both cases the values for the acid counterpart (“D”, “E”) were considered for the calculation of the protein properties. Biovenn was used for Venn diagrams [37].

## Results and discussion

### Qualitative and quantitative aspects of the DTR versus FASP protocols

**Fig. 2:**
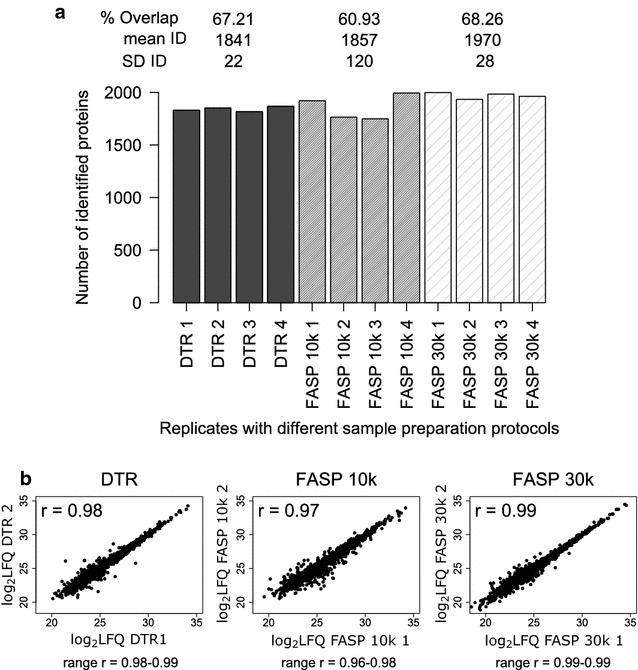
Qualitative and Quantitative reproducibility for the DTR and FASP protocol. **a** Numbers of identified Proteins (ID) for each replicate of the three protocols DTR (black), FASP 10 k (grey) and FASP 30 k (shaded). For each protocol, the mean and standard deviation (SD) of the ID as well as the proteome overlap were calculated. **b** Log2 transformed label-free quantitation values (LFQ) of the four replicates per protocol were plotted against each other and the Pearson correlation coefficient (r) was calculated. The plots and Pearson correlation coefficients of the first two replicates are shown while for the other correlations the range of the obtained coefficients were given for each protocol

We analyzed four single murine kidney tissue slices either with the DTR protocol or with the FASP workflow using 10 and 30 k filter devices. The sample preparation with the DTR protocol was more straightforward and faster, as the centrifugation steps make the FASP protocols lengthy and labor-intensive. The performance of the DTR protocol ranged between the two FASP protocols, showing high protein overlap and Pearson correlation coefficients and only slightly lower numbers of identified proteins. In a single, non-fractionated LC–MS/MS analysis the DTR protocol identified on average 1841 ± 22 proteins whereas slightly higher numbers were found with the FASP 10 and 30 k approach, namely 1857 ± 120 and 1970 ± 28 proteins (Fig. 2a). The proteins identified with the FASP 10 k protocol showed the lowest overlap of only 61% within the four replicates, whereas the overlap of the DTR and FASP 30 k protocol were 67 and 68% (Fig. 2a, Additional file 1A). For all three methods, about 10% of the proteins were found in only one replicate, another 10% in two replicates and another 10% in three out of four replicates (Additional file 1B). The peptide coverage per protein was similar for the three techniques. Proteins found in all four replicates were represented on average by 12 peptides, while proteins, which were found in less replicates, were represented by only one to three peptides (Additional file 1C). This highlights that proteins with a higher sequence coverage are more consistently found across replicates.

**Fig. 3:**
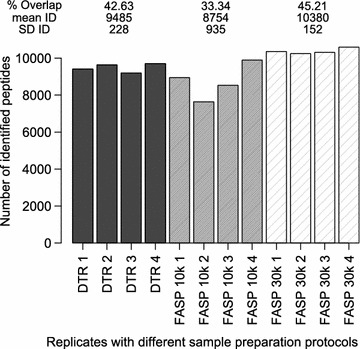
Peptide numbers and overlap in the four replicates. Numbers of identified peptides (ID) for each replicate of the three protocols DTR (black), FASP 10 k (grey) and FASP 30 k (shaded). For each protocol, the mean and standard deviation (SD) of the ID as well as the proteome overlap were calculated

On the peptide level the FASP 10 k protocol identified 8754 ± 935 unique peptides compared to 9485 ± 228 unique peptides for the DTR protocol and 10,380 ± 152 for the FASP 30 k protocol. The peptide overlap between replicates was 33% with the FASP 10 k protocol and 43 and 45% with the DTR and FASP 30 k protocol (Fig. 3, Additional file 2A).

The higher yield and better reproducibility for the 30 k compared to the 10 k filter has been reported before and many FFPE studies based on the FASP protocol made use of the 30 k filter [9, 38–41]. The centrifugation with the 10 k filter took three times longer than with the 30 k filter as the filter was frequently clogged by tissue debris. Due to the filter clogging for the FASP protocol and the more straightforward approach of the DTR protocol, it is not surprising to obtain a lower peptide yield in the BCA assay for the FASP protocols after tryptic digestion (Additional file 2B). The higher peptide yield with the DTR protocol does not correlate with higher identification rates as the same peptide amount was injected into the mass-spectrometer, but it might enable sample fractionation, which would lead to a better proteome coverage.

Just like protein identification, protein quantitation need to be reproducible in order to perform reliable quantitative proteomics studies. We found stable normalized LFQ intensities across all replicates (Additional file 3). All Pearson correlation coefficients were > 0.95 and confirm high quantitative reproducibility for all three approaches. FASP 30 k showed the highest correlation with all coefficients being 0.99, closely followed by DTR with 0.98–0.99 and lastly FASP 10 k with coefficients between 0.96 and 0.98 (Fig. 2b). In Additional file 4 proteome overlaps and Pearson correlation coefficients between all replicates are depicted.

**Fig. 4:**
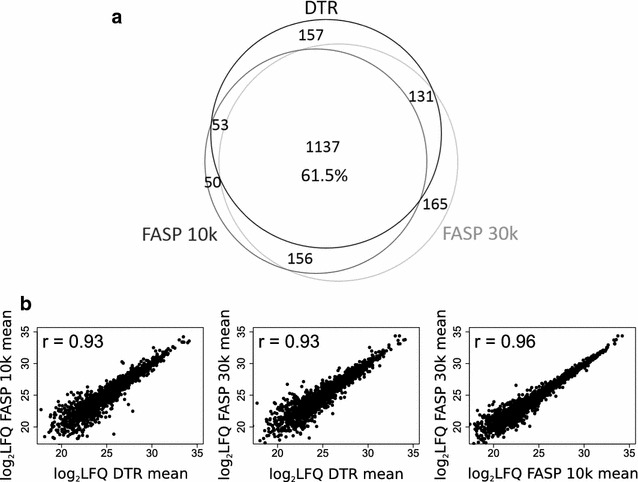
Comparison of identified and quantified proteins between the three protocols. **a** Proteins identified in all four replicates per protocol were compared for their proteome overlap. The Venn diagram depicts the numbers of proteins shared between the DTR (black), FASP 10 k (dark grey) and FASP 30 k (light grey) protocols. **b** The mean protein abundances were calculated for each protocol based on the log2 transformed label-free quantitation values (LFQ) and plotted against each other. Shown are also the corresponding Pearson correlation coefficients

**Fig. 5:**
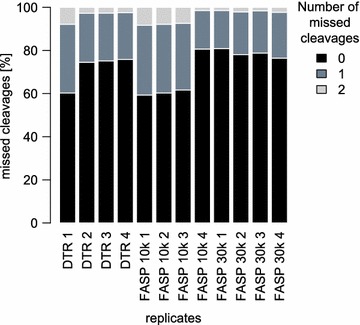
Numbers of missed cleavages for all replicates. For each replicate, the percentages of missed cleavages are plotted

Comparing the different protocols between each other shows that they share 1137 proteins (61.5%), when considering only proteins identified in all four replicates (Fig. 4a). The partially incomplete overlap of proteome coverage is expected for protocols using different sample preparation steps and extraction buffers with varying physicochemical properties. In addition it is also known to be an intrinsic feature of mass-spectrometry based proteomics per se [42]. In terms of LFQ intensities, the DTR and FASP protocols correlate with a decent Pearson correlation coefficient of 0.93, while the FASP protocols between themselves correlate with a slightly higher coefficient of 0.96 (Fig. 4b). The DTR protocol shows a reproducible 15% increase in missed cleavages compared to the FASP protocol (Fig. 5). These missed cleavages do not substantially affect quantitation as seen by high Pearson correlation coefficients between the replicates of the different protocols.

### Physicochemical and functional aspects of the identified proteins

We furthermore assessed different physicochemical properties and Gene Ontology (GO) term distributions to exclude enrichment of proteins with extreme properties by the DTR protocol. In all analyses, we observed similar profiles between proteins of the DTR and FASP approaches.

**Fig. 6:**
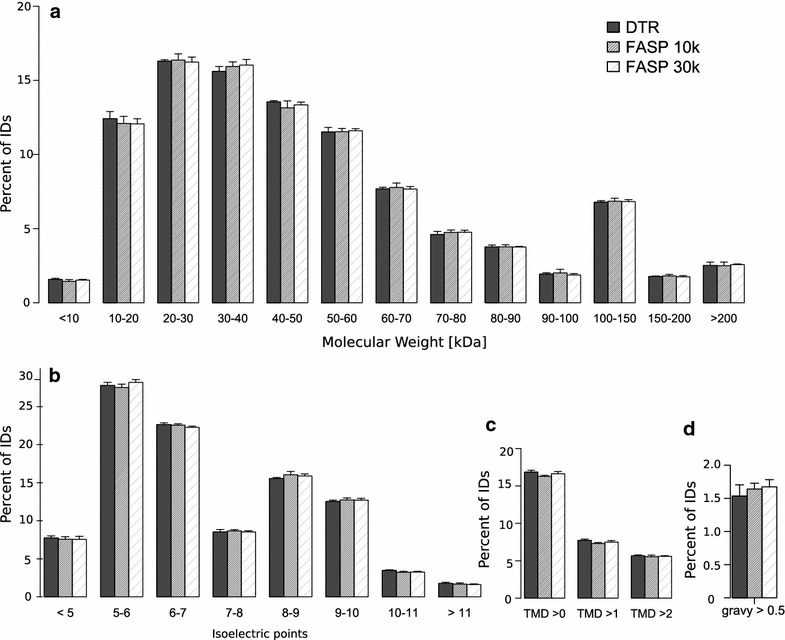
Physicochemical properties of the proteins identified with each protocol. Distribution of the Molecular weight (**a**) isoelectric point (**b**) gravy > 0.5 (**c**) and transmembrane domains (**d**) of the identified proteins. Mean and standard deviation of the four replicates for the DTR (black), FASP 10 k (grey) and FASP 30 k (shaded) are shown

The molecular weight distribution does not show major differences between the protocols (Fig. 6a). About 70% of the proteins have a molecular weight between 10 and 60 kDa and less than 3% of proteins have a molecular weight smaller than 10 kDa, showing no difference between the protocols as the small proteins are retained in the filter devices during the FASP approach because they are unfolded (Fig. 6a) [38]. The protocols do not differ substantially in high molecular weight proteins in contrast to a study from Tanca et al. [41] which reported an increase in high molecular weight proteins with a detergent free direct trypsinization approach compared to FASP 30 k.

There was also no major difference between the distribution of the proteins isoelectric points, the abundance of proteins with a gravy score > 0.5 or the number of estimated transmembrane domains (Fig. 6b–d). This means that the proteins show no major differences in terms of their charge or their hydrophobic properties, validating the physicochemical similarity of the two detergents RapiGest and SDS. The physicochemical similarity between detected proteins with the direct trypsinization protocol compared to the FASP approach can be lost when omitting the RapiGest in the extraction buffer as reported by Tanca et al. [41]. On the other hand, Drummond et al. [22] compared the direct trypsinization protocol in the presence or absence of acid-labile surfactant and reported similar proteome coverage with a high overlap concluding that it is preferable to use no detergent in order to prevent losses while removing it. The basis of these conflicting results remains unclear. In the present study, we performed RapiGest removal in the presence of 3 M guanidinium hydrochloride to prevent sample losses.

**Fig. 7:**
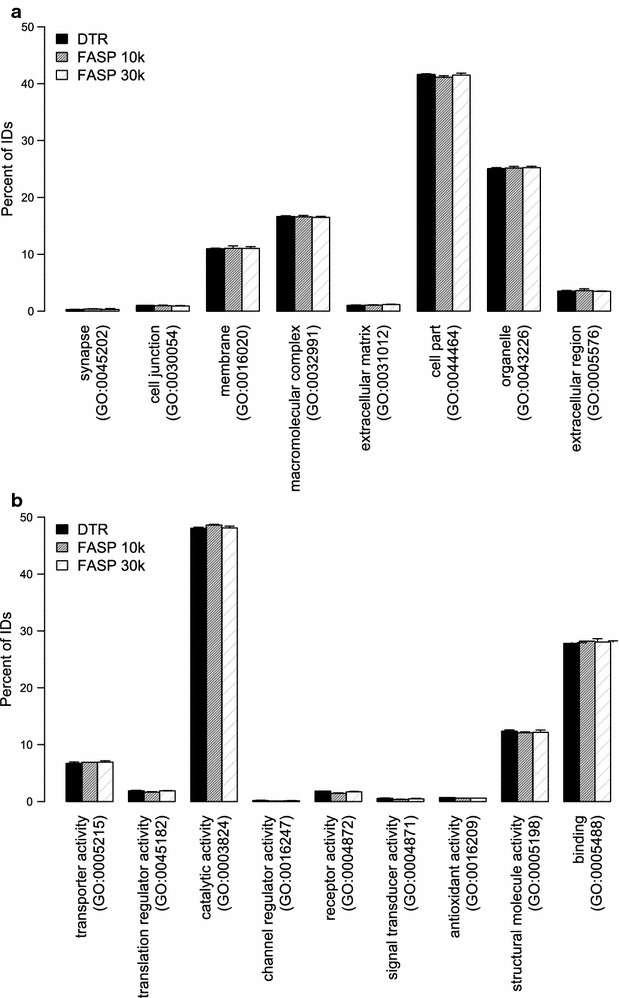
Distribution of gene ontology (GO) terms of identified proteins. Distribution of the identified proteins according to the cellular compartment (**a**) and to the molecular function (**b**) gene ontology terms. Mean and standard deviation of the four replicates for the DTR (black), FASP 10 k (grey) and FASP 30 k (shaded) are depicted

Gene ontology terms for cellular compartment and molecular function were assigned to the proteins using PANTHER. The distribution of the percentage of proteins belonging to each GO term is plotted in Fig. 7. The distribution of cellular compartment and molecular function GO terms is highly similar for the three different protocols (Fig. 7a, b). Proteins identified in all protocols show around 41% of the proteins assigned to be “cell part” and around 11% to be membrane associated. In terms of their function, 48% of them have catalytic activity while the second largest group with 28% are binding proteins.

We conclude that the DTR and FASP protocols yield highly comparable outcome and are both applicable to single FFPE tissues slices. In our study, each slice consisted of 0.47–0.6 mm^3^ tissue. We did not titrate down the tissue amount to keep the conditions suitable for both protocols. Although FASP is reported to be usable for small sample amounts as little as 500 laser micro dissected cells [9, 40, 43] several studies consider it non-ideal for small tissue samples [20, 22, 44]. Three recent publications highlighted the need for robust protocols that can be applied to smaller FFPE tissue specimens and two of them used the direct trypsinization approach, one with and the other without Rapigest [20, 22, 45].

### Compatibility of histological staining methods with the DTR protocol

**Fig. 8:**
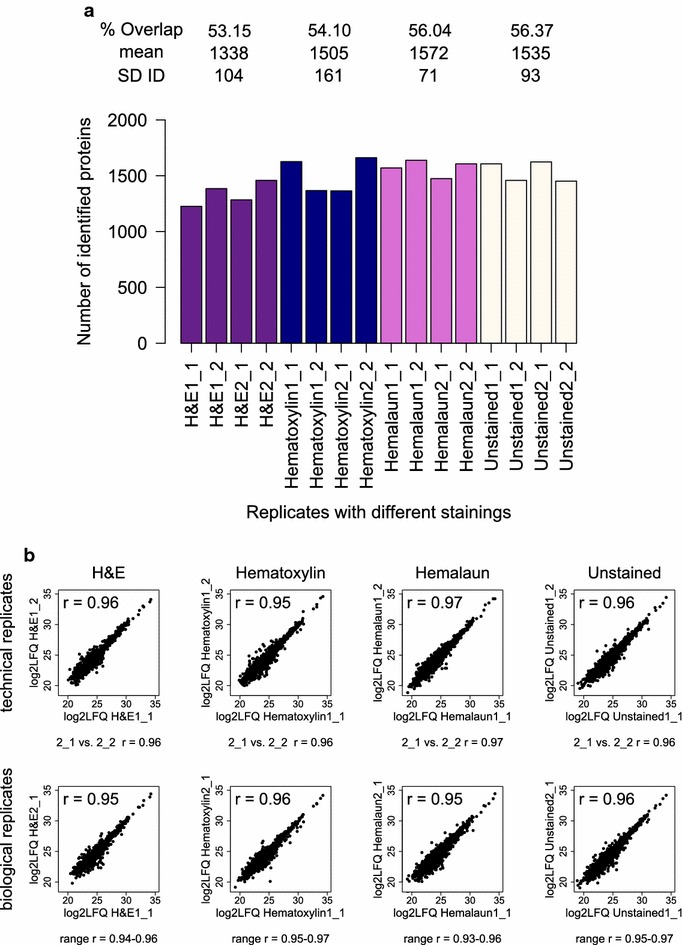
Qualitative and Quantitative reproducibility for differently stained FFPE tissues. **a** Numbers of identified Proteins (ID) for each replicate of the different histological stained tonsil tissues with H&E (purple), Hematoxylin (blue), Hemalaun (pink) and unstained (white) are shown. For each staining, the mean and standard deviation (SD) of the ID as well as the proteome overlap were calculated. X_Y refers to the number of biological (X) and technical replicate (Y). **b** Log2 transformed label-free quantitation values (LFQ) of the four replicates per staining were plotted against each other and the Pearson correlation coefficient (r) was calculated. The plots and Pearson correlation coefficient of the first two replicates for technical and biological replicates are shown while for the other correlations the range of the obtained r-values is given for each staining method

We stained four single FFPE tonsil tissue slices, derived from two patients, with different standard histological stainings and analyzed them with the DTR protocol. More than 1500 proteins were on average identified from the tissues in each analysis, comprising unstained slices or slices stained with hematoxylin and hemalaun. H&E stained tissues led to average protein identifications of only 1338 ± 104 (Fig. 7a). The overlaps of identified proteins were slightly lower for the H&E and hematoxylin stained tissue than for the hemalaun stained tissue and unstained control (53.2 and 54.1% compared to 56 and 56.4%). The LFQ intensities were distributed equally for all replicates (Additional file 5). Pearson correlation coefficients of LFQ protein intensities were > 0.93 for all replicates with the same staining showing that label-free quantitation strategy was not impaired (Fig. 8b). As expected, Pearson correlation coefficients are slightly higher in the comparison of tissue slices from the same patient than from the two different patients. Additional file 6 depicts the proteome overlaps and Pearson correlation coefficients between all replicates.

**Fig. 9:**
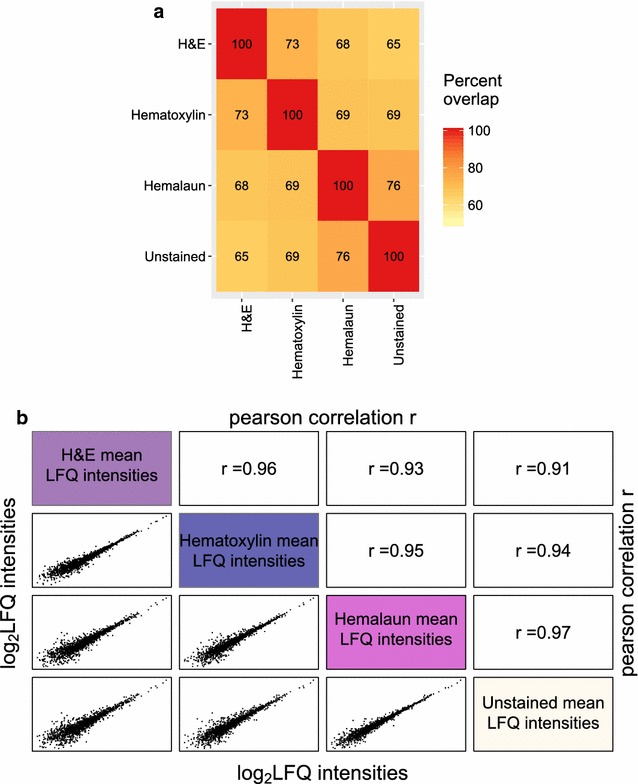
Comparison of identified and quantified proteins between the four different staining methods. **a** Proteins identified in all four replicates per staining method were compared for their proteome overlap. The heat map depicts the percentage of proteins, which were shared between two staining methods. **b** The mean protein abundances were calculated for each staining method based on the log2 transformed label-free quantitation values (LFQ) and plotted against each other. Shown are also the corresponding Pearson correlation coefficients

The comparison of the three different staining methods and the unstained control illustrates that staining does not severely impair proteome coverage and quantitation, as suggested by similar proteome overlap of identified proteins in all four replicates and correlations of mean protein quantitation values (Fig. 9a, b). Hemalaun stained tissues specimens shared the highest percentage of commonly identified proteins (76%) and the best Pearson correlation coefficient (0.97) with the unstained control tissue specimens. In this respect, hematoxylin stained tissue specimen came second (69% and 0.94) and H&E stained tissue third (65% and 0.91). Interestingly, this “ranking” correlates with the staining durations, which are part of the routine protocols of the Institute for Surgical Pathology, University Medical Centre Freiburg, in detail: 1 min for hemalaun, 4 min for hematoxylin and 4 min hematoxylin plus 1 min eosin for H&E staining. Our study aimed to investigate compatibility of commonly used histopathological staining procedures with the DTR method and it remained beyond the scope of the present study to probe whether shortened incubation times yield further improved proteome coverage. Nevertheless, by choosing incubation times in the 1–4 min range, our results underline robustness of the DTR technique and suggest its usability for archived samples. In other studies, hematoxylin has been found to be compatible with mass-spectrometry proteomics [39, 46]. But a very short staining time of only 20 s was suggested to increase proteome coverage [46]. On the other hand, despite staining for only 10 s with hematoxylin, Becker et al. [47] reported a 50% decrease in protein yield after extraction with the Qproteome FFPE Tissue Kit. For fresh- or snap frozen tissues, there are contradictory reports about the influence of H&E staining on the protein yield and recovery in gel-based proteomics, which seemed to be mainly influenced by the dehydration steps performed after staining, which is not applied to FFPE tissue [48–51]. In summary, there is no consensus perspective on the impact of histopathological staining on proteome coverage. However, in many situations there is the need to source archived specimens for retrospective proteome studies, irrespective of any procedural details of their staining. Our results encourage considering such specimens for proteome studies. When possible, it might be beneficial to perform a small pretest to find the optimal staining method.

The compatibility of stained FFPE tissue with the DTR protocol might be astonishing as no dedicated cleanup step is performed during sample preparation. Our findings go along with a study by Drummond et al. [22] in which, immuno- and cresyl staining did not interfere with the numbers and overlap of proteins when applying a direct trypsinization protocol without detergent. Recently, Azimi et al. [21] were able to use H&E stained tissue with the DTR protocol for mass-spectrometry analysis.

We conclude that DTR is compatible with commonly used tissue staining procedures, thus opening the possibility to use previously stained tissues for proteomic analysis. We propose when possible to prefer hemalaun and hematoxylin to the most common staining method H&E, to keep staining times as short as possible and to first perform a small pilot experiment when using different tissues.

## Conclusions

In essence, the DTR protocol performed almost as well as FASP. We found that the DTR protocol can keep up with the FASP protocol in terms of qualitative and quantitative reproducibility. Despite detecting 15% more missed cleavages in the DTR protocol, the protein quantitation was comparable to the FASP approach and the proteome coverage was only slightly decreased. The protocols showed no major difference for the physicochemical properties and GO terms annotations of the detected proteins. We showed that tissues stained with H&E, hematoxylin and hemalaun are compatible with the DTR protocol. Our study strengthens previous studies where DTR was used and underlines that DTR is a viable alternative to FASP.

## Additional files

**Additional file 1.** Shared proteins between the replicates and peptide numbers per protein. (A) Overlap between identified proteins. (B) Frequency of shared proteins between the replicates. (C) Average peptide numbers for each protocol and proteins depending on their appearance in multiple replicates.

**Additional file 2.** Shared peptides between replicates and peptide amounts. (A) Peptide overlap between replicates. (B) Peptide amounts measured by BCA assay after tryptic digestion.

**Additional file 3.** Distribution of LFQ intensities for the DTR and FASP protocols. Log2 transformed LFQ intensity distribution depicted for all replicates with DTR in black, FASP 10 k in dark grey and FASP 30 k in light grey.

**Additional file 4.** Proteome overlaps and Pearson correlation coefficients between all DTR and FASP replicates. (A) Percent of shared proteins between all replicates. (B) Pearson correlation coefficients of LFQ intensities between all replicates.

**Additional file 5.** Distribution of LFQ intensities for the differently stained tissues processed with the DTR protocol. Distribution of log2 transformed LFQ intensities for all replicates stained with H&E (purple), hematoxylin (blue), hemalaun (pink) or unstained (white).

**Additional file 6.** Proteome overlaps and Pearson correlation coefficients between the differently stained tissues prepared with the DTR protocol. (A) Percent of shared proteins between all replicates. (B) Pearson correlation coefficients for the correlation of LFQ intensities between all replicates.

## Abbreviations

DTR: direct tissue trypsinization with RapiGest
DTT: dithiothreitol
FASP: filter aided sample preparation
FFPE: formalin-fixed paraffin-embedded
LC–MS/MS: liquid chromatography–tandem mass spectrometry
H&E: hematoxylin & eosin
HIAR: heat induced antigen retrieval
LFQ: label-free quantitation
SDS: sodium dodecyl sulfate
