# Supplementary figures and images for: Reproducible proteomics sample preparation for single FFPE tissue slices using acid-labile surfactant and direct trypsinization
Source: Clin Proteomics. 2018 Mar 6;15:11. doi: 10.1186/s12014-018-9188-y (PMC5838928; doi:10.1186/s12014-018-9188-y)

**A**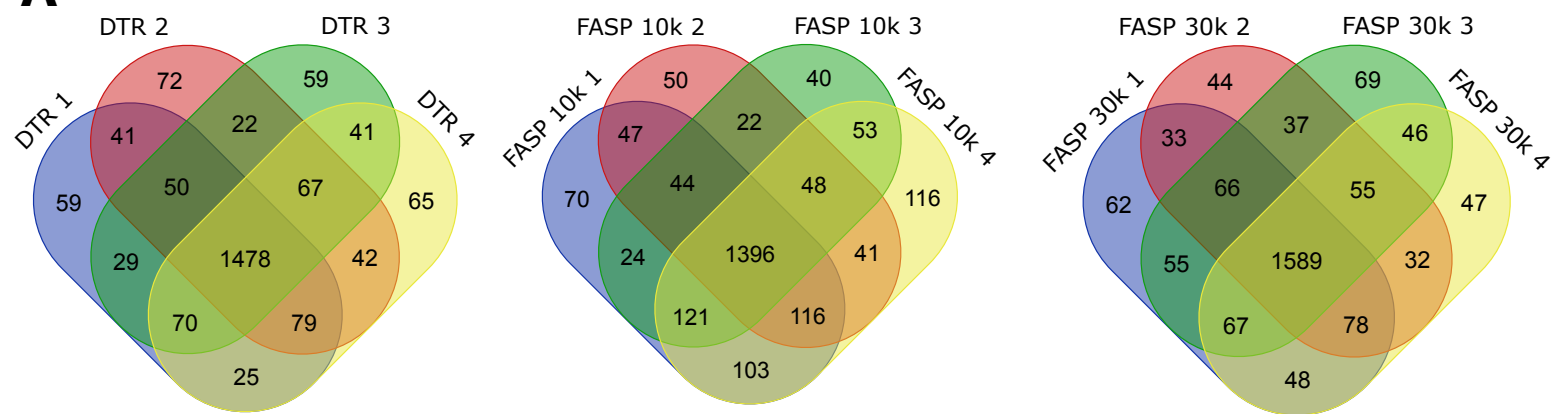**B**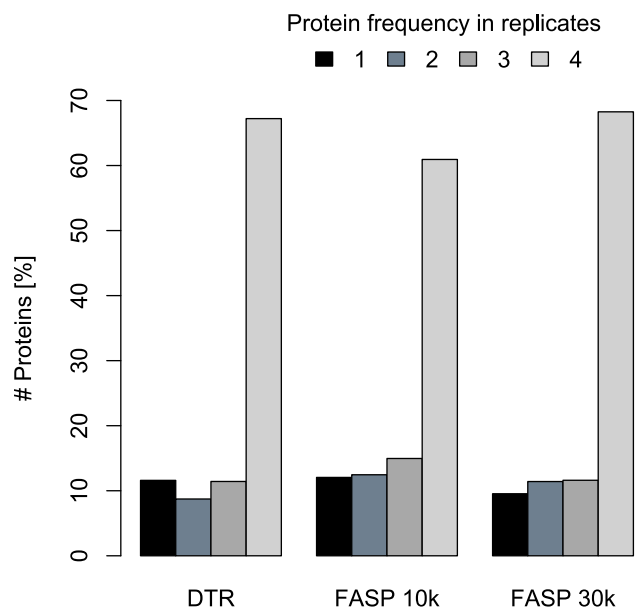**C**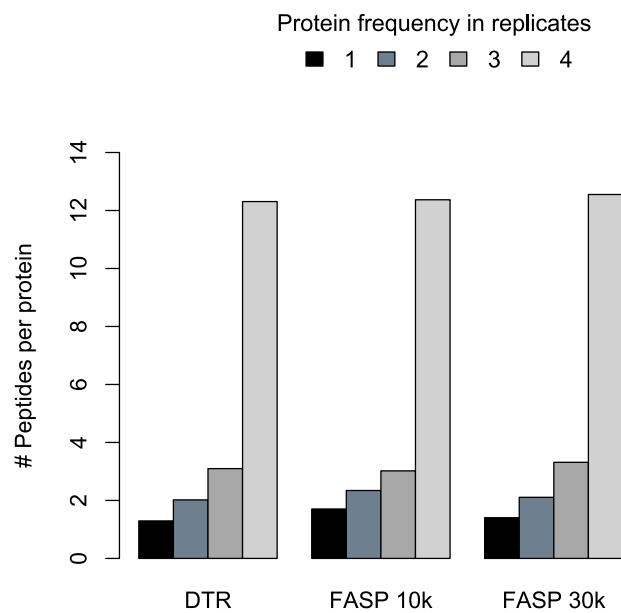

Supplement: Supplementary file 1 — Additional file 1. Shared proteins between the replicates and peptide numbers per protein. (A) Overlap between identified proteins. (B) Frequency of shared proteins between the replicates. (C) Average peptide numbers for each protocol and proteins depending on their appearance in multiple replicates. [file 12014_2018_9188_MOESM1_ESM.pdf]

**A**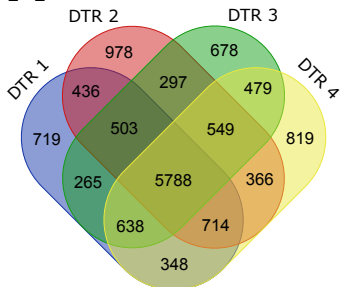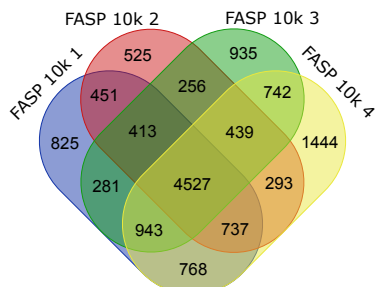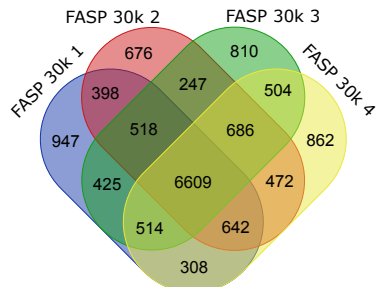**B**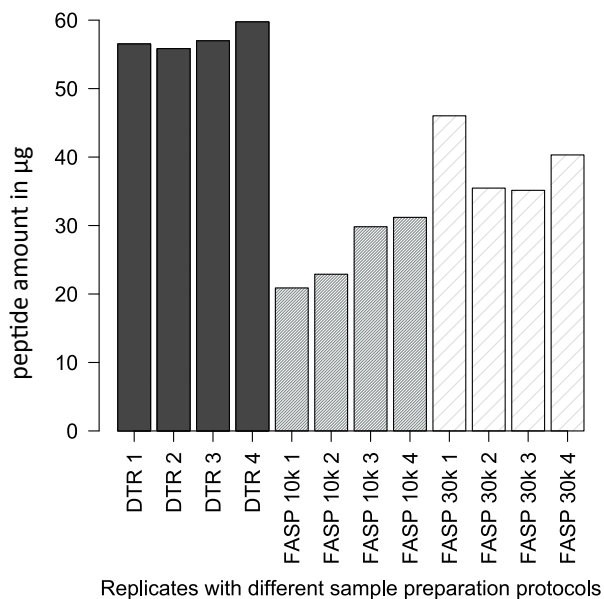

Supplement: Supplementary file 2 — Additional file 2. Shared peptides between replicates and peptide amounts. (A) Peptide overlap between replicates. (B) Peptide amounts measured by BCA assay after tryptic digestion. [file 12014_2018_9188_MOESM2_ESM.pdf]

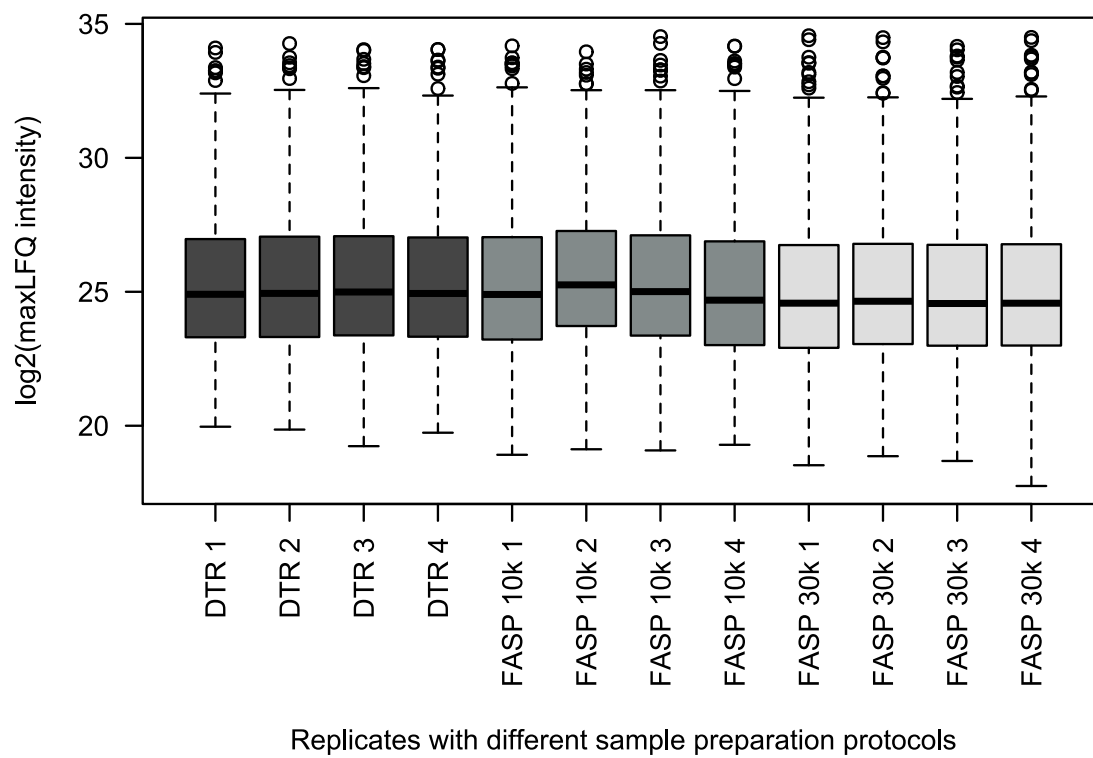

Supplement: Supplementary file 3 — Additional file 3. Distribution of LFQ intensities for the DTR and FASP protocols. Log2 transformed LFQ intensity distribution depicted for all replicates with DTR in black, FASP 10 k in dark grey and FASP 30 k in light grey. [file 12014_2018_9188_MOESM3_ESM.pdf]

**A**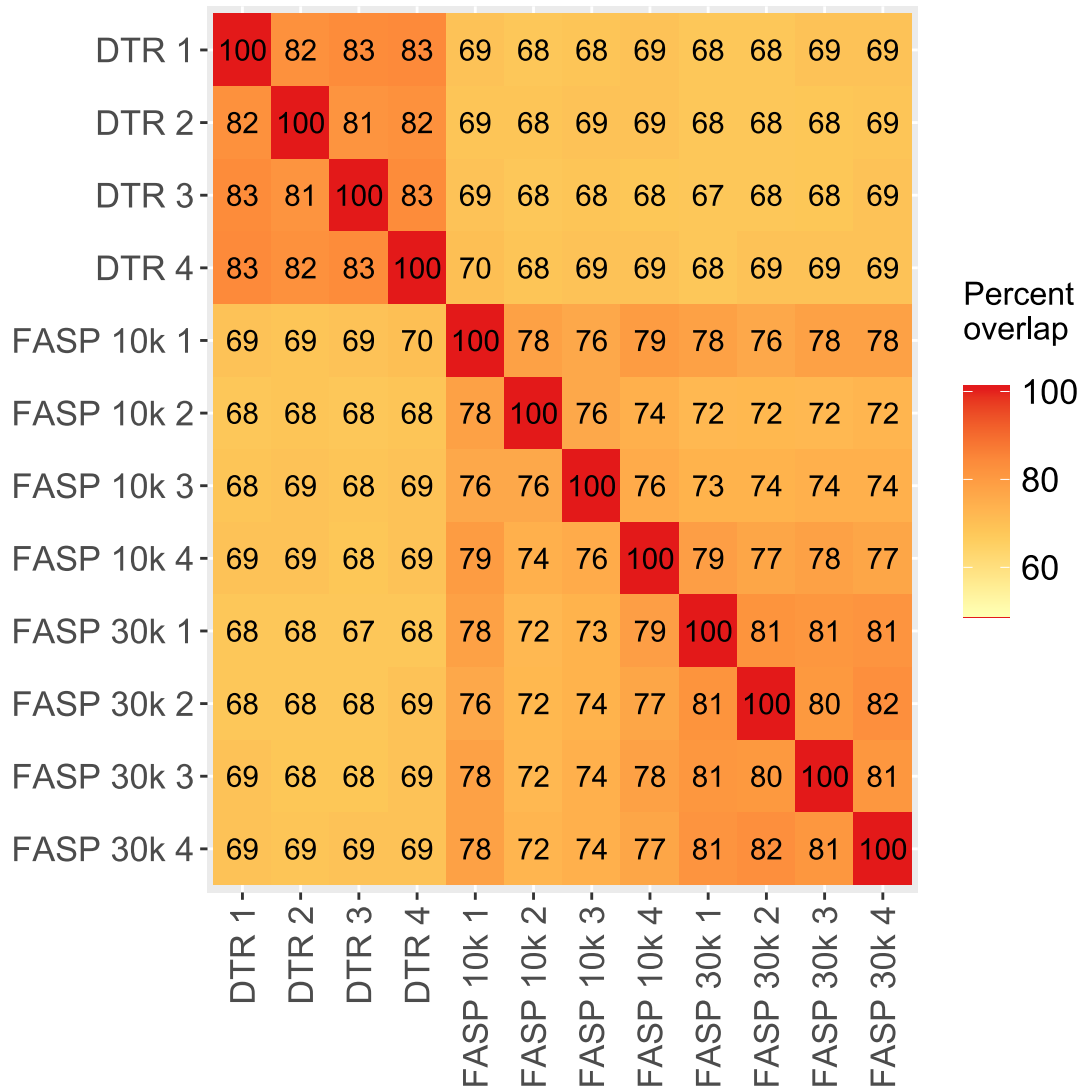**B**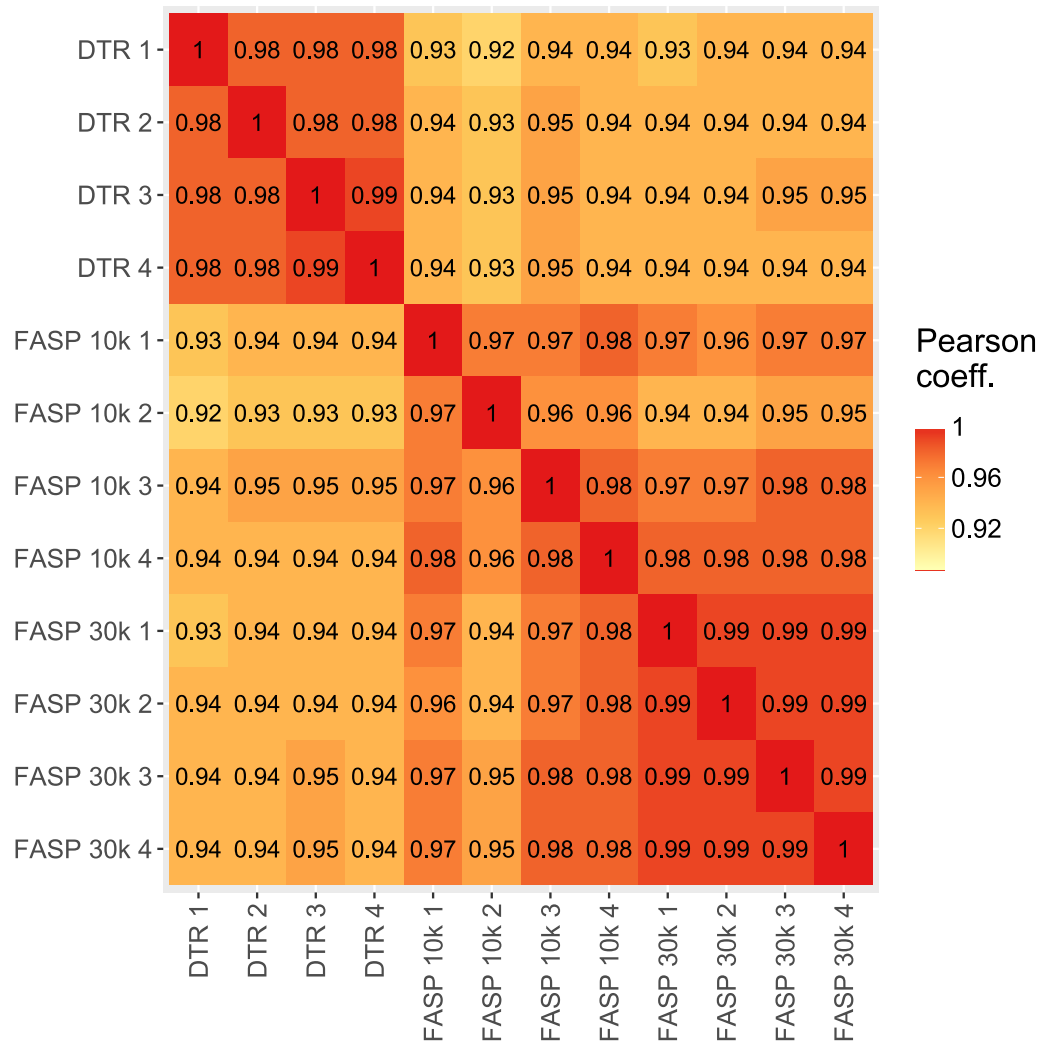

Supplement: Supplementary file 4 — Additional file 4. Proteome overlaps and Pearson correlation coefficients between all DTR and FASP replicates. (A) Percent of shared proteins between all replicates. (B) Pearson correlation coefficients of LFQ intensities between all replicates. [file 12014_2018_9188_MOESM4_ESM.pdf]

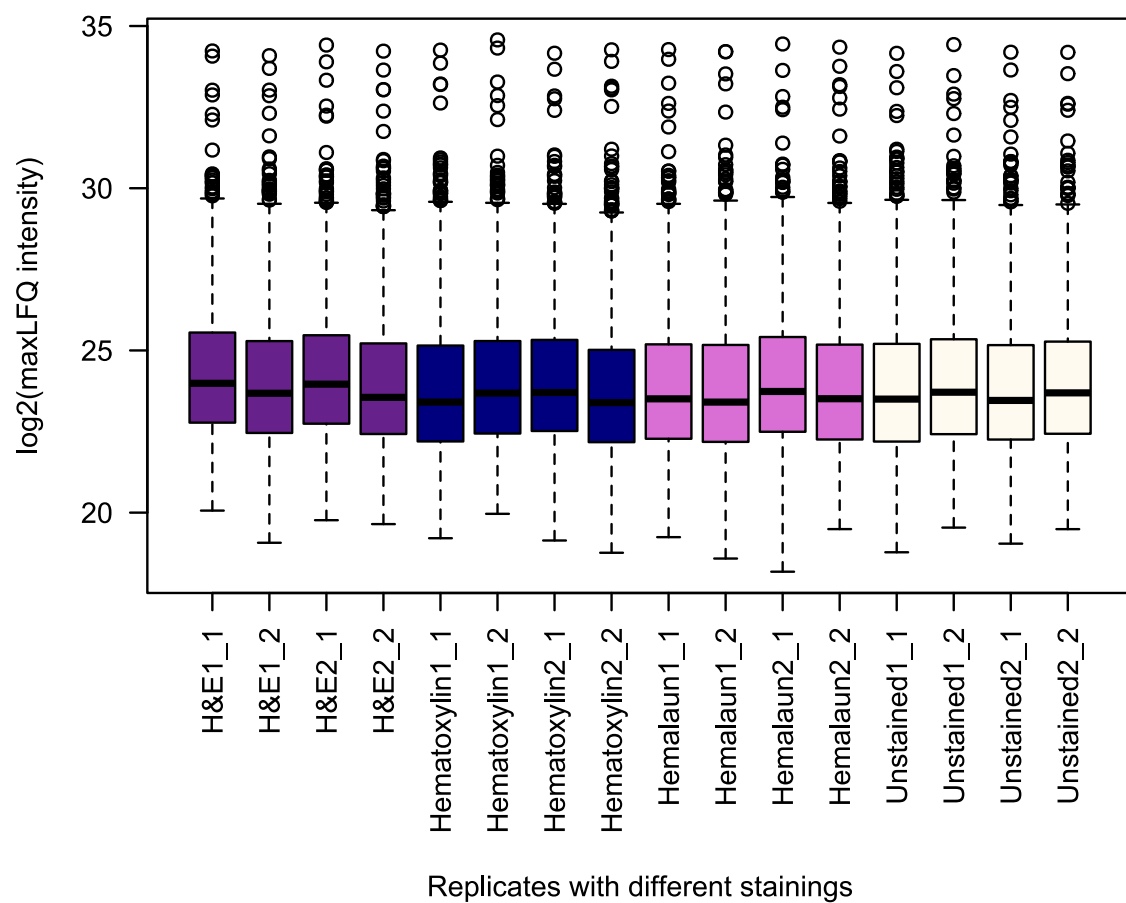

Supplement: Supplementary file 5 — Additional file 5. Distribution of LFQ intensities for the differently stained tissues processed with the DTR protocol. Distribution of log2 transformed LFQ intensities for all replicates stained with H&E (purple), hematoxylin (blue), hemalaun (pink) or unstained (white). [file 12014_2018_9188_MOESM5_ESM.pdf]

**A**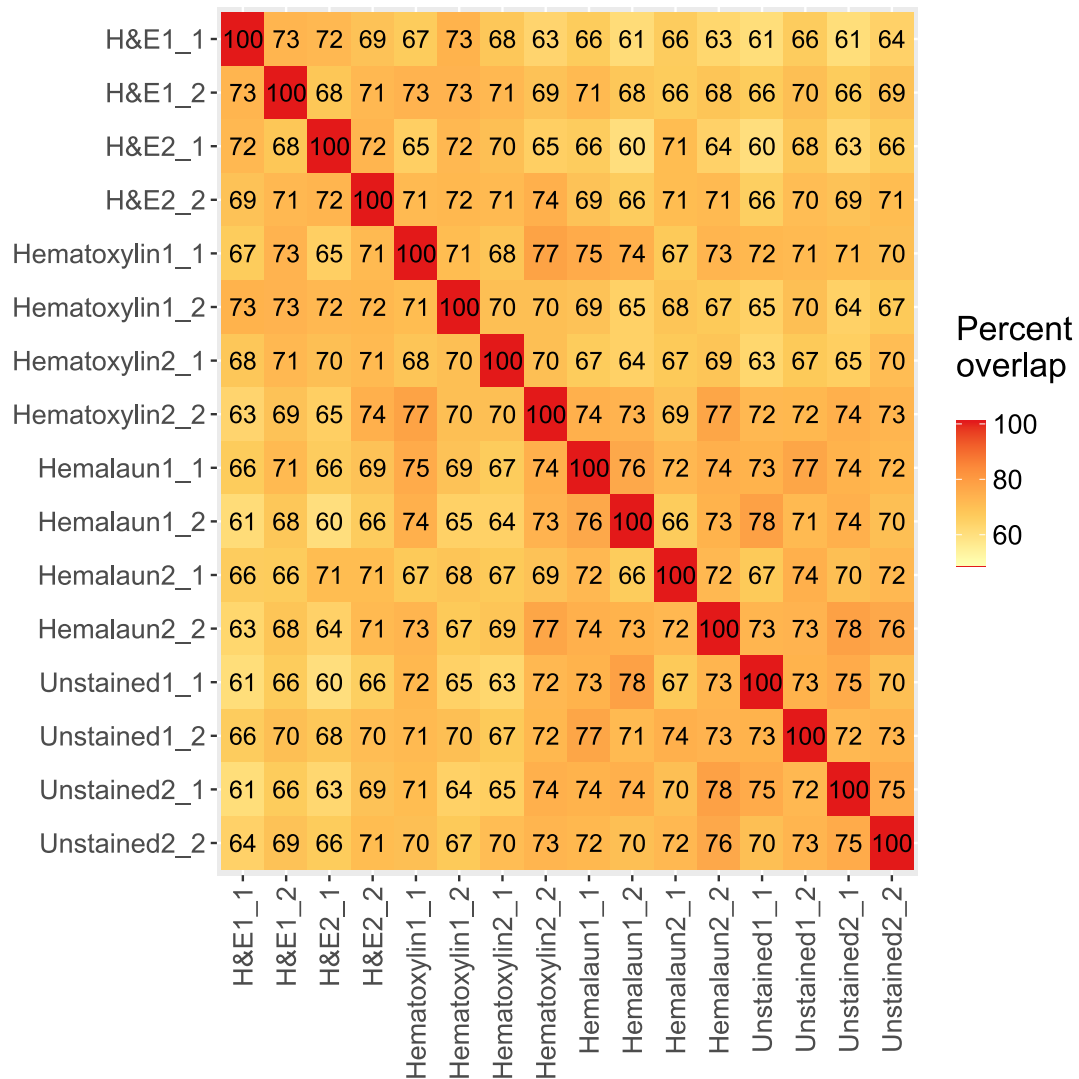**B**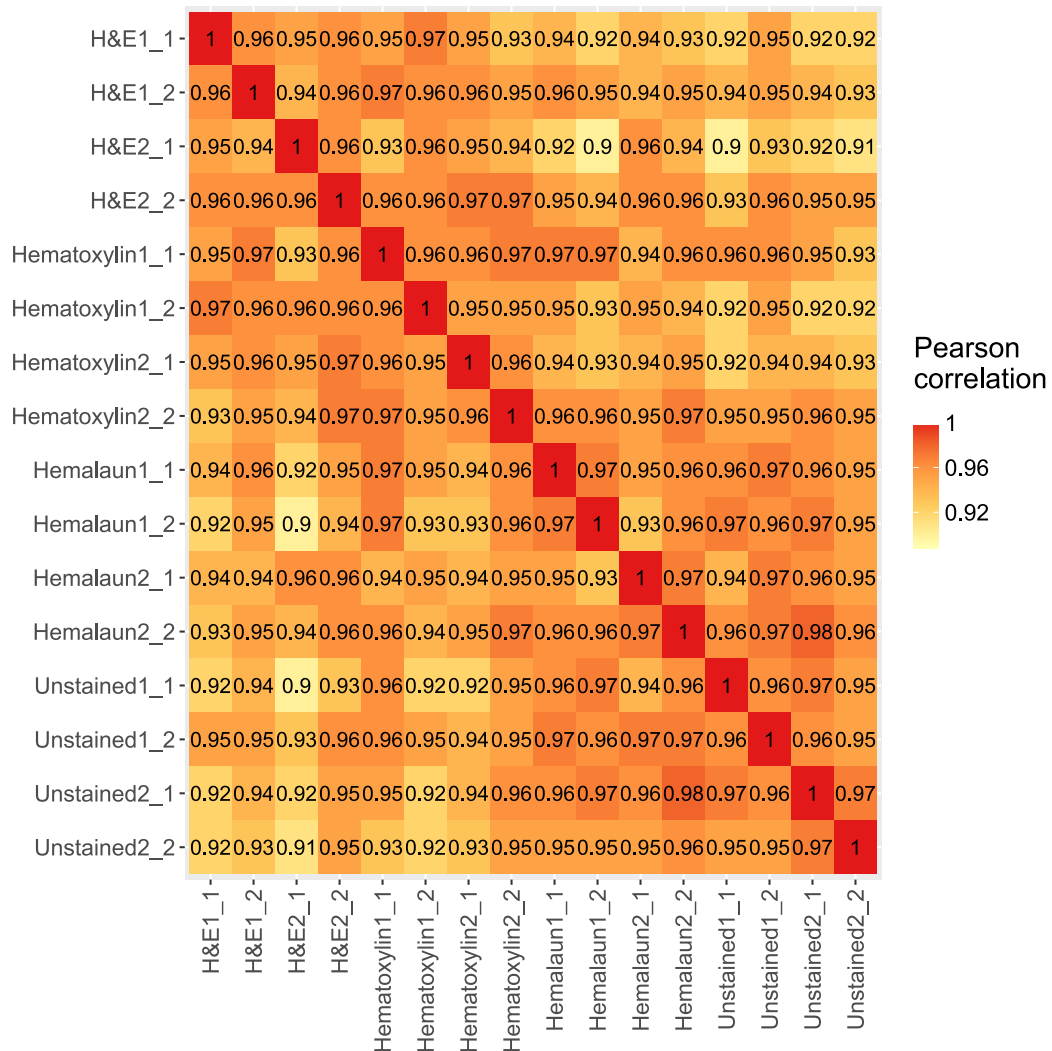

Supplement: Supplementary file 6 — Additional file 6. Proteome overlaps and Pearson correlation coefficients between the differently stained tissues prepared with the DTR protocol. (A) Percent of shared proteins between all replicates. (B) Pearson correlation coefficients for the correlation of LFQ intensities between all replicates. [file 12014_2018_9188_MOESM6_ESM.pdf]
